# Effectiveness of government strategies for financial protection against costs of hospitalization Care in India

**DOI:** 10.1186/s12889-018-5431-8

**Published:** 2018-04-16

**Authors:** Alok Ranjan, Priyanka Dixit, Indranil Mukhopadhyay, Sundararaman Thiagarajan

**Affiliations:** 1 School of Health Systems Studies, Tata Institute of Social Sciences, Mumbai, India; 2 School of Health Systems Studies, Tata Institute of Social Sciences, Mumbai, India; 3 School of Government and Public Policy, O P Jindal Global University, Sonipat, Haryana India

**Keywords:** Universal health coverage (UHC), Public funded health insurance (PFHI), Out-of-pocket expenditure (OOPE), Catastrophic health expenditure (CHE)

## Abstract

**Background:**

In the past decade, India has seen the introduction of many ‘publicly funded health insurance’ schemes (PFHIs) that claim to cover approximately 300 million people and are essentially forms of purchasing care from both public and private providers to reduce out-of-pocket expenditure (OOPE) for hospitalization.

**Methods:**

Data from a recent government-organized nationwide household survey, The National Sample Survey 71st Round, were used to analyse the effectiveness and equity of tax-funded public health services and PFHIs as distinct but overlapping approaches to financial protection for hospitalization across different socio-economic categories. Cross-tabulation analysis, multivariate logistic regression and propensity score matching were the main analytical methods used.

**Results:**

Government hospitals provide access to 45.6% of all hospitalization needs. Although poorer quintiles use public hospitals more often, even in the poorest quintile, as many as 37.2% are utilizing private hospitals. The average OOPE that a household experiences for hospitalization in public hospitals is approximately only one-fifth of the OOPE for hospitalization in the private sector. PFHI schemes cover 12.8% of the population, and coverage is higher in upper quintiles and in urban areas. Hospitalization rates increase with PFHI coverage, and this occurs with both public and private providers. Propensity score matching shows that PFHI contributes to a marginal reduction (1%) in ‘catastrophic health expenditure incidence at the 25% threshold’ (CHE-25) for the bottom three quintiles. The reported coverage of PFHIs was greater in the upper income quintiles. Utilization of public services was greater in the poorer income quintiles and more marginalized social groups.

**Conclusions:**

Periodic surveys are essential to guide policy choices regarding the appropriate mix of strategies for financial protection in pluralistic systems. There is a need for caution regarding any shift in the role of governments from providing services to purchasing care, given the contexts and limitations of currently available PFHIs. Even with tax-funded public services, although the average OOPE is lower than the care purchased through PFHIs, there is still a modest level of CHE and impoverishment due to health care costs that persist. Both strategies need to be synergized for more effective financial protection.

**Electronic supplementary material:**

The online version of this article (10.1186/s12889-018-5431-8) contains supplementary material, which is available to authorized users.

## Background

The emergence of universal health coverage (UHC) as the dominant discourse in global health brought financial protection against direct household payments for health care to centre-stage [1]. The formal definition provided in the glossary of the first monitoring report is elaborate and comprehensive: “Universal health coverage means all people receiving the health services they need, including health initiatives designed to promote better health (such as anti-tobacco policies), prevent illness (such as vaccinations), and provide treatment, rehabilitation and palliative care (such as end-of-life care) of sufficient quality to be effective while at the same time ensuring that the use of these services does not expose the user to financial hardship” [2]. Though financial protection forms a crucial aspect of the definition of UHC, such financial protection need not be equated only with insurance schemes. Tax-financed free care or care subsidized by public health facilities receives either superficial mention or is ignored altogether when discussing financial protection [3], even though it has all the elements of pre-payment and risk-pooling [4].

In the period after 2005, state and central governments in India launched a number of Public Funded Health Insurance (PFHI) programmes. These are not social health insurance as commonly understood. The central government’s Rashtriya Swashtya Bima Yojana (RSBY) programme (translates to National Health Insurance Scheme) is meant to cover all those below the poverty line, but individual families have to pay a token sum each year to enrol or renew membership, and such enrolment and renewal is not mandatory [5]. The insurance agencies provide the enrolment or renewal, and the government pays a premium for each household that is covered. In most of the state schemes, coverage is conferred on those who are eligible based on income criteria that are certified by the public distribution system, which distributes income category cards to enable lower socioeconomic groups to access subsidized food grains. All of these PFHI schemes provide for cashless services at the point of care for a wide list of hospitalizations [6–8]. They do not provide coverage for ambulatory care [9]. In contrast to these post-2005 PFHIs, there are two earlier social insurance schemes that are operational from the 1950s – one for regular government employees and another for organized workers (Employees State Insurance Scheme or ESI), and they cover ambulatory care as well, in which enrolment and contribution are mandatory. The package of services is larger, and the sum assured are also much higher. However, these two schemes account for only approximately 35 million of the 300 million stated as covered under all PFHIs [10]. We have described the key features of the PFHIs in Additional file 1.

In the period leading up to the finalization of the 12th Five Year Plan, there were pressures within the Indian Planning Commission to shift the role of government from a provider to a purchaser of services, including from an integrated network of providers. After a new government came to power in 2014, the Niti Aayog, an institution that supplanted the Planning Commission, continued with this policy thrust. This shift was contested [11]. The recently announced National Health Policy 2017 calls for both strategies and provisions of tax-funded free care by public health hospitals and primary care facilities as well as an expansion of strategic purchasing using PFHIs [12]. However, there are concerns about where the emphasis would be in regard to implementation [13, 14].

This paper aims to discuss a) the coverage and effectiveness of both government purchasing through insurance and government provision of tax-funded free or subsidized care as strategies of financial protection; b) the contribution that PFHI makes to the reduction in catastrophic health expenditures due to hospitalization; and c) the equity dimensions of both financial protection strategies. Since government purchasing of health care is currently limited almost exclusively to hospitalization, we have assessed financial protection strategies only with reference to hospitalization expenses. We also discuss the potential of this National Sample Survey (NSS) health survey and database to be one of the central tools to measure progress and guide policy with regard to charting India’s roadmap towards UHC.

## Methods

The data for the paper are derived from the unit records of the “Social Consumption: Health” survey (71st round) conducted by the NSS Office, which provides information at national and state (provincial) levels related to morbidity, hospitalization, reported causes for illness and hospitalization, the cost of care, the type of service provider, and care in pregnancy and for the elderly [15].

In India, the NSS Office, a government institution, organizes annual surveys on different areas of consumption, poverty, and employment, and once every 10 years, it undertakes a survey called Social Consumption: Health [15].

This survey uses a two-stage stratified sampling approach, with the first sampling units composed of village and urban blocks and the second stage composed of households. Data collection was performed from January 2014 to June 2014 in two segments of 3 months each. A total of 65,932 households (rural: 36480, urban: 29452) were surveyed for the entire Indian Union, which included a total of 333,104 individuals (rural: 189573, urban: 143531; male: 168697 female: 164407).

Morbidity as reported by the household is captured in response to three questions: 1) Were you ill in the past 15 days? 2) Were you hospitalized in the past 365 days? 3) If so, what was the cause? The reported cause is then attributed to the nearest fit of one of 60 diagnostic categories on the basis of a medical diagnosis conveyed to the study team by the respondent, or failing this approach, the main symptom was used. This survey also probed whether each household and individual had any insurance coverage, including the type of insurance coverage. For each illness or hospitalization, the study team noted the choice of provider and reported costs of care, disaggregated into medical and non-medical costs by component. The sample consisted of 57,456 hospitalization episodes (55,026 episodes excluding death), of which approximately 10,168 episodes had insurance coverage.

Those with insurance coverage were asked about the type of scheme, and their responses were categorized into four groups. One is the PFHIs, which include both the social insurance schemes that began in the 1950s and the post-2005 government-sponsored schemes that target the poorer socio-economic groups for coverage. The second category is composed of individual households that voluntarily purchase their own insurance coverage from private insurance firms or “private insurance”. The third category is employer-provided insurance coverage for employees, and a fourth category of “others” is associated with special schemes such as Yashwasini, which is privately initiated and linked to cooperatives. Because the last is very small, we have not shown it in the calculations (only 0.1%). Of these four types of insurance, only PFHI and the post-2005 types of schemes focus on providing financial protection for the poor and are being considered for scaling up as the national strategy. Therefore, we have limited most of our analysis to only this first category, and within that, we separately analysed the lower three quintiles since those covered by the two social insurance schemes would largely or entirely belong to the upper two quintiles.

This study examines various dimensions of equity, defined as unequal insurance coverage, hospital rates, and/or financial protection based on sex, social group, economic quintile, or rural/urban residence. This study measures financial protection in terms of seven indicators. These are 1) the mean OOPE per hospitalization episode; 2) the median OOPE per hospitalization episode; 3) the proportion of hospitalization episodes where OOPE was less than Rs 500; 4) the proportion of hospitalization episodes where OOPE was less than Rs 1000; 5) the incidence of households experiencing catastrophic health expenditures, which is the proportion of households whose costs of hospitalization were beyond a threshold, defined as 10% of annual consumption expenditure (CHE-10); 6) the incidence of catastrophic health expenditure, which is the proportion of households whose costs of hospitalization exceeded a threshold, defined as 25% of annual consumption expenditure (CHE-25); and 7) the impoverishment related to hospitalization costs.

OOPE for hospitalization was calculated per episode, including transportation, and reimbursements were subtracted. Only 2% of those hospitalized received reimbursements.

The two indicators of incidence of hospitalization for which OOPE was below Rs 500 or Rs 1000 are introduced since the stated objective of most government-funded insurance schemes is to provide cashless services for hospitalization care, not merely a reduction in OOPE. Since some incidental expenses may be counted, thresholds of Rs 500 and Rs 1000 for OOPE enabled better measurement of the proportion of hospitalizations in which this objective of cashless services was achieved.

The methodology discussed by Wagstaff and Doorslaer [16] to assess catastrophic health expenditure (CHE) for healthcare was applied in this study. A household OOPE on hospitalization in the preceding year is considered an incidence of CHE when the payment exceeds the 10% (CHE-10) and 25% (CHE-25) thresholds of the household annual total household consumption expenditure. This household annual total consumption expenditure represented 12 times the UMPCE (usual monthly per capita consumption expenditure). UMPCE is measured by a consumption survey using a methodology standardized by NSSO and accepted widely in India as a reliable proxy for income levels. We chose to study OOPE and CHE only in regard to hospitalization because this is what approximately all government insurance covers.

To estimate impoverishment due to hospitalization costs, this study used the threshold recommended by the Government of India Planning Commission Report of June 2016 for measuring the poverty line (per capita) as Rs 972 in rural and Rs 1407 in urban areas [17].. Households whose MPCE was initially above this threshold but later fell below it after incurring hospital expenditures were considered to have experienced ‘impoverishment due to hospitalization costs’. Households whose MPCE was initially itself below the poverty line were considered to have experienced ‘deepening of poverty due to hospitalization costs’.

The levels of financial protection are measured and presented for four situations: a) care is from the private sector, and there is no insurance coverage; b) care is from the private sector, and there is PFHI coverage; c) care is from a tax-funded public provider, which affords financial protection through subsidies, but there is no insurance coverage; and d) care is from the tax-funded public provider and is complemented by PFHI coverage. This allows us to compare the reduction in OOPE and the protection against CHE that are provided in each of these contexts.

Other than the above, another indicator of financial hardship available from this survey is the source of financing. Households were asked for the source of financing for hospitalization, and responses were categorized into following options: 1) household income or savings; 2) borrowings; 3) sale of physical asset; 4) contributions from friends and relatives and 5) other. There could be multiple responses. Having to borrow or sell physical assets as one of the sources of financing indicates financial hardship.

We know that enrolment in PFHI is not random, and it is correlated with the OOPE and CHE; those who were enrolled in PFHI may differ from those who were not in some systematic way. In this situation, reduction in OOPE and CHE could be under-estimated because of confounding factors, such as economic quintile, type of provider, social group, education level, sex and disease category for which treatment was sought.

We used propensity score matching (PSM) to match for these characteristics across the households *with* PFHI coverage to those *without* any insurance coverage to estimate the contribution that PFHI makes to reducing the incidence of CHE due to hospitalization expenses. In cross-sectional data, PSM establishes that an intervention of interest (in this case PFHI) contributes to an outcome of interest (in this case household CHE incidence). This method ensures that other observed background characteristics or variables are matched in intervention and non-intervention groups so that their influence can be controlled. Using a counterfactual model, we have estimated the average outcome of the treated households (which is the incidence of CHE in households with insurance coverage in this study) and the average outcome that the treated households would have obtained in the absence of PFHI, which is unobserved. The average treatment on treated (ATT), which measures the average difference in CHE incidence that PFHI affords to households with PFHI coverage, is a measure of the effectiveness of the PFHI [18].

We matched for the following six variables: sex, social group (caste), education of head of household, economic quintile, choice of public or private provider and disease category. The main causes of hospitalization were categorized into 12 groups: infections, cancers, metabolic and blood diseases, mental and neurological illness, eye, cardiovascular, respiratory, gastro-intestinal, musculo-skeletal and genito-urinary, obstetric (including childbirth), injuries and others. The nearest neighbour matching method with replacement was used in combination with a logit model. To satisfy the balancing property on all of the background characteristics, a ‘hit’ or ‘miss’ approach was used.

The category of PFHI includes a variety of schemes (see Additional file 1), including those that provide insurance coverage for government employees. To study the contribution of the subset of PFHIs that are designed to prevent CHE among the poorer socio-economic groups of the society, we have conducted the PSM in two stages. First, PSM was applied to the total population (all five income quintiles), and later, it was applied only for the bottom three quintiles. We conducted PSM for all hospitalizations, irrespective of quintile groups, then conducted PSM again separately for the bottom three quintiles.

This study also examined whether PFHI coverage is associated with a change in the choice of provider since PFHIs were expected to overcome financial barriers to access of care in the private sector. Furthermore, to also study whether PFHI coverage led to increased utilization of hospitalization services, a multiple logit model was constructed, with the likelihood of hospitalization as the dependent variable and with publicly funded insurance coverage, education, age, economic quintile, sex, urban residence and social status as independent variables. Analysis was conducted using STATA software (version 13).

## Results

The findings are presented below in the form of replies to the following four broad questions:

- What are the extent and effectiveness of financial protection provided by tax-funded public provisioning of health care services alone? To what extent is this coverage equitable?
- What are the extent and effectiveness of public purchasing of health care using PFHIs? Effectiveness in this context is based on reductions in OOPE, increases in cashless hospitalization and a decreasing incidence of catastrophic health expenditure and impoverishment due to hospitalization.
- How does government purchasing through insurance complement government provisioning of health care to provide effective financial protection?
- Do government-funded insurance schemes increase hospitalization rates or shift care provision from the public to the private sector?

### Financial protection through tax-funded public providers

Public hospitals provide hospitalization services to 45.4% of all hospitalized patients; in rural areas, this increases to 50.4% (see Additional file 2). The average OOPE in a public hospital for someone who has no insurance is Rs 3994 (USD 67) or approximately one-fifth of the average OOPE in a private hospital (Rs 20,445 or USD 341) (see Table 1).

**Table 1:** Choice of provider and average OOPE (the median) with PFHI coverage and no insurance

	No insurance			Those with government insurance		
UMPC -Rural	% of total hospitalization cases treated in public hospital	Average OOPE in Public	Average OOPE in Private	% of total hospitalization cases treated in public hospital	Average OOPE in Public	Average OOPE in Private
All	50.8	3994	20,445	49.8	2848	17,493
Poorest	67.7	2934	16,281	79.0	2175	17,480
Poor	61.7	3958	16,581	62.7	2828	11,892
Middle	52.6	3733	14,975	56.8	2735	17,846
Rich	47.4	3882	18,470	40.2	2553	17,378
Richest	29.1	6834	28,364	34.3	3871	18,756
UMPC -Urban						
All	36.1	6322	27,102	40.4	2738	19,111
Poorest	51.6	2901	17,525	57.6	1886	13,129
Poor	42.0	5492	20,200	47.8	1606	16,905
Middle	33.6	5902	23,768	38.6	4293	17,672
Rich	23.3	16,929	36,493	35.5	3262	18,705
Richest	16.2	13,241	39,865	24.4	2614	26,009

Among those belonging to the poorest quintile, the proportion of hospitalized patients treated in public hospitals increases to 67.7% in rural areas and 51.6% in urban areas compared to 29.1% in rural areas and 16.2% in urban areas among the richest quintile (see Table 1). In the sense that utilization occurs relatively more frequently in the poorer economic quintiles, access to the public hospital is equitable.

However, even in the poorest quintile in rural areas, almost one-third (32.3%) use the private sector despite it being much costlier. The average OOPE per hospitalization in public hospitals increases with income quintile, from Rs 2934 for the poorest to Rs 6834 for the richest in rural areas and from Rs 2901 to Rs 13,201 in urban areas (see Table 1).

However, average household costs for accessing public hospitals are much lower compared to the costs for accessing private hospitals. This does not necessarily prevent catastrophic health expenditures (16.2% at CHE 10 and 6.22% at CHE 25), impoverishment (6.49% increase post hospitalization) and ‘poverty deepening’ for those already below the poverty line (see Tables 2 and 3, last row).

**Table 2:** Catastrophic Health Expenditure (percentage of households where OOPE on hospitalization exceeding the 10% or 25% threshold of total annual consumption expenditure)

	CHE > 10% threshold	CHE > 25% threshold	N (Households)
Total	39.62	18.22	45,261
Geographical location			
Rural	39.04	18.09	24,858
Urban	40.84	18.52	20,403
Insurance schemes			
Government funded	38.31	17.87	6604
Employer supported	29.59	12.88	651
Arranged by household	31.67	13.91	713
Not covered	40.17	18.48	37,157
Total	39.62	18.22	45,261
Social groups			
Schedule Tribes	25.72	10.23	5412
Schedule castes	35.66	15.91	7633
Other backward classes	41.29	19.31	17,934
Others	43.52	20.32	14,282
UMPC quintile (rural)			
Poorest	36.21	16.98	4694
Poor	34.45	16.92	4318
Middle	37.34	15.59	5178
Rich	38.48	17.65	5187
Richest	47.18	22.58	5480
UMPC quintile (urban)			
Poorest	37.85	18.58	4982
Poor	39.32	18.65	4760
Middle	43.58	17.59	4372
Rich	45.82	20.91	3266
Richest	37.93	16.91	3020
Service provider			
Private	60.74	29.04	22,742
Public	16.19	6.22	22,519

**Table 3:** Impoverishment effect of OOPE on hospitalization

	Percentage of household below poverty line pre-payment	Percentage of household below poverty line post-payment	N (Households)
Total	27.13	39.86	45,261
Geographical location			
Rural	27.37	40.38	24,858
Urban	26.61	38.77	20,403
Insurance schemes			
Government funded	21.85	33.51	6604
Employer supported	11.04	17.33	651
Arranged by household	3.53	10.33	713
Not covered	28.83	42.01	37,157
Total	27.13	39.86	45,261
Social groups			
Schedule Tribes	42.83	52.35	5412
Schedule castes	35.82	47.35	7633
Other backward classes	26.31	39.38	17,934
Others	18.22	31.90	14,282
UMPC quintile (rural)			
Poorest	100.0	100.0	4694
Poor	43.49	76.11	4318
Middle	0.00	20.27	5178
Rich	0.00	10.44	5187
Richest	0.00	5.41	5480
UMPC quintile (urban)			
Poorest	100.00	99.97	4982
Poor	20.20	53.41	4760
Middle	0.00	10.80	4372
Rich	0.00	9.37	3266
Richest	0.00	3.95	3020
Service provider			
Private	18.56	36.93	22,742
Public	36.63	43.12	22,519

### The extent and effectiveness of PFHI

The survey shows that 15.2% of the population of India is covered under some kind of insurance (see Table 4), an estimated 190 million people (the population in 2014 was 1.25 billion). Of the various kinds of insurance schemes, PFHIs had the highest coverage (12.8%), indicating that 160 million people were covered, a number that is significantly lower than official estimates [10]. Only 1.2% of the population possesses insurance coverage arranged by households themselves via private insurance, with another 1.2% covered through employers. We limit our analysis to PFHIs.

**Table 4:** Coverage rates (in percent) of different insurance schemes by various stratifiers (among households surveyed)

Strata	No coverage	Public Funded Health Insurance (PFHI)	Any coverage^*^	N (Individuals)
Total	84.8	12.8	15.2	333,104
Geographical location				
Rural	85.9	13.1	14.1	189,573
Urban	82.0	12.0	18.0	143,531
Sex				
Male	85.0	12.5	15.0	168,697
Female	84.5	13.1	15.5	164,407
Social groups				
Schedule Tribes	81.0	18.3	19.0	43,142
Schedule castes	86.1	13.1	13.9	55,454
Other backward classes	84.5	13.6	15.5	133,565
Others	85.6	9.5	14.4	100,943
UMPC quintile (rural)				
Poorest	88.7	10.6	11.3	44,499
Poor	88.2	11.2	11.8	35,516
Middle	87.9	11.4	12.1	40,335
Rich	82.6	16.5	17.4	35,890
Richest	80.2	17.7	19.8	33,333
All	85.9	13.1	14.1	189,573
UMPC quintile (urban)				
Poorest	90.4	8.6	9.6	43,372
Poor	87.5	10.7	12.5	34,404
Middle	81.4	13.9	18.6	28,834
Rich	76.9	14.1	23.1	20,729
Richest	63.6	15.1	36.4	16,192
All	82.0	12.0	18.0	143,531

Note: (^*^): Any coverage is the sum of government funded and employee supported insurance coverage, arranged by household and other insurance schemes

#### Equity in PFHI coverage

Coverage of PFHI is marginally higher in urban areas compared to rural areas (13.1% to 12.0%). Insurance coverage by sex is approximately equal – not a surprising finding, as most insurance schemes have households as the units of coverage (see Table 4). Although most of the PFHIs are meant for the poor, insurance coverage is higher among the wealthy: the coverage among the lowest and highest quintiles is 10.6% and 17.7% in rural areas and 8.6% and 15.5% in urban areas, respectively (see Table 4).

#### Reduction in OOPE

Mean OOPE on hospitalization in the preceding year was lower among those covered under PFHIs (Rs 10,943) compared to those who are not covered (Rs 14,436) (see Additional file 3). The stated objective of most government-funded insurance schemes is, however, to provide cashless services for hospitalization care, not merely a reduction in OOPE. Only 2.8% of hospitalizations among the insured received cashless services compared to 1.5% among those without insurance. We also find that 70% of those with government insurance and 79.4% without insurance spent more than Rs 1000.

#### PFHI and catastrophic health expenditure

Of those households which reported hospitalization in the preceding year, 39.62% of households incur CHE-10, and 18.22% of households incur CHE-25. The catastrophic expenditure for hospitalization did not vary with urban or rural location (see Table 2).

The proportion of households experiencing hospitalization who incurred CHE-10 is highest among the uninsured (40.2%), and among those covered with PFHI, it is marginally lower (38.2%). Using CHE-25, it is 18.5% for the uninsured and 17.9% for those with PFHI coverage.

Although savings are the main source of financing for most people, approximately 20.1% of hospitalization cases reported borrowing as the first source of financing. Another 34.1% reported borrowing as a second source (see Additional files 4 and 5).

Considering CHE by social group, the proportion of households experiencing CHE increases with higher social status and with increasing income quintile (see Table 2). This could be due to changes in choice of provider, poorer patients limiting their level of expenditure or differential pricing.

Results from the PSM for all quintile groups show that for the unmatched sample estimate, those households that had PFHIs were only 5% less likely to experience CHE-10 compared to households that had no insurance coverage (see Table 5). The calculated ATT values in the treated and control groups were 0.36 and 0.49, respectively, which means that after matching, 36% of PFHI households had CHE-10 compared to 49% of non-insured households. The estimates based on a second model indicate that 16% of PF HI households had experienced CHE-25 compared to 23% of non-insured households, a difference of 6% (see Table 5).

**Table 5:** Impact Assessment of PFHI on CHE at 10% and 25% threshold using Propensity Score Matching (PSM)

	Public insurance Vs. No Insurance	Treated	Controls	Difference	S.E.	T-test	P > z	95% confidence interval
Model A (10% CHE)	Unmatched	0.36	0.41	−0.05	0.01	−7.13		
	ATT	0.36	0.49	−0.13	0.02^*^	−5.15	0.00^*^	−0.16, −0.10^*^
	ATU	0.41	0.41	0.01				
	ATE			−0.01				
Model B (25% CHE)	Unmatched	0.16	0.19	−0.02	0.01	−4.71		
	ATT	0.16	0.23	−0.06	0.01^*^	−3.21	0.00^*^	−0.09, − 0.04^*^
	ATU	0.19	0.20	0.01				
	ATE			−0.00				

Note: ^*^ based on Bootstrap Standard Error

*ATT* Average Treatment on Treated, *ATU* Average Treatment on Untreated, *ATE* Average Treatment Effect

However, when the PSM was conducted for the bottom three quintiles separately, the results show that for the unmatched sample estimate, households who have PFHI are 2% and 1% less likely to experience CHE-10 and CHE-25, respectively (see Table 6). After matching, 36.6% PFHI households experience CHE-10 compared to 37% of non-insured households (ATT is 0.366 and 0.3699, respectively). The estimates for CHE-25 indicate that 18% of non-PFHI households incur CHE-25 compared to 17% of PFHI households in the bottom three quintile groups. This means that for the bottom three quintile groups, PFHI is responsible for a 0.4% and 1% difference in CHE-10 and CHE-25, respectively. Thus, the effectiveness of PFHIs with respect to catastrophic health expenditure is reduced further when we consider only the bottom three quintiles.

**Table 6:** Impact Assessment of PFHI on CHE at 10% and 25% threshold using Propensity Score Matching (PSM) for below three quintiles

	Public insurance Vs. No Insurance	Treated	Controls	Difference	S.E.	T-test	P > z	95% confidence interval
Model A (10% CHE)	Unmatched	0.366	0.385	−0.02	0.009	−2.17		
	ATT	0.366	0.37	−0.004	0.03^*^	−0.14	0.00^*^	−0.04 to − 0.001
	ATU	0.385	0.345	−0.04				
	ATE			−0.036				
Model B	Unmatched	0.171	0.179	−0.008	0.007	−1.17		
(25%	ATT	0.171	0.181	−0.01	0.027^*^	−0.38	0.00^*^	−0.022 to 0.005
CHE)	ATU	0.179	0.163	−0.015				
	ATE			−0.015				

Note: ^*^ based on Bootstrap Standard Error

*ATT* Average Treatment on Treated, *ATU* Average Treatment on Untreated, *ATE* Average Treatment Effect

#### Impoverishment due to OOPE for hospitalization

Approximately 27.13% of households were poor before hospitalization (see Table 3). When hospitalization expenditures are incurred, the proportion of households below the poverty line (BPL) increases to 39.86%. In rural India, this figure increases from 27.37% (pre-hospitalization) to 40.38% (post-hospitalization), and in urban India, it increases from 26.61% to 38.77%. The PFHIs did not act as a protection against impoverishment as households still incurred significant hospitalization costs.

We note that among those who were already BPL before being hospitalized, there is further deepening of poverty due to expenses of hospitalization, and in those marginally above the poverty line, the hospitalization pushes them below the line. In the lowest quintile, where all are below the poverty line by definition, their depth of poverty can only intensify. In the second lowest quintile group, approximately 44% in rural areas and 20% in urban areas are BPL. After hospitalization, the amount is 76% and 53%, respectively. Households that belong to the rest of the quintiles are above the poverty line to start with. In the middle group, 20.27% fall below poverty after hospitalization. Even in the top two rural quintiles, 10.4% and 5.4% are impoverished due to hospitalization (see Table 3).

### PFHI interactions with private and public provisioning

Table 7 shows the effectiveness of financial protection with different combinations of PFHI and choice of provider using 7 distinct indicators of financial hardship. There is clearly a gradient. In the private hospital with PFHIs, both the mean and median OOPE decrease (see Additional file 6), but this has minimal impact on catastrophic health expenditures and on impoverishment. In all seven indicators, subsidized care in the public hospital, even without insurance, leads to far less financial hardship compared to care from private providers, even with insurance. When tax-funded public provisioning is complemented by PFHI coverage, there is a further modest increase in the effectiveness of financial protection for hospitalization in public hospitals by all indicators of financial protection. This synergy leads to a 34% decrease in the mean and median OOPE, a 45% increase in incidence of hospitalization where OOPE was less than Rs 500 and a modest decrease in CHE-10 (8%) and CHE-25 (7%) (comparing the last two columns of Table 7).

**Table 7:** Financial Protection with PFHI: Interaction of Coverage and Choice of Provider

	Private provider without any insurance	Private provider with PFHI	Public provider without any insurance	Public provider with PFHI
Mean OOPE per hospitalization	22,604	17,741	4919	3204
Median OOPE per hospitalization	11,300	10,120	1451	950
% of hospitalization episodes with OOPE< 500 Rs.	1.4	6.6	27.4	39.8
% of hospitalization episodes with OOPE< 1000 Rs.	2.6	9.7	41.6	53.1
Incidence of CHE-10	62.4	60.0	16.1	14.8
Incidence of CHE-25	30.0	29.2	6.0	5.6
Impoverishment	19.1	18.2	6.8	4.6

### PFHI, hospitalization rates and choice of provider

In rural areas, the proportion of households choosing to use a public versus private provider is approximately the same, regardless of PFHI coverage status, while in urban areas, there is a 4% increase in public sector utilization (see Table 1). Overall, (irrespective of insurance) 45.4% of hospitalization episodes were treated by various public providers, whereas the remaining 54.6% cases were treated by private providers (see Additional file 2). Disaggregating by income quintile, the results (see Table 1) show that across urban and rural areas and across approximately all quintiles, there is a small increase in utilization of a public hospital with insurance coverage. Poorer quintiles in both urban and rural areas access public services at over twice the rate of the richest, and this proportion does not change with PFHI coverage.

The hospitalization rate is 4.2% for households with no coverage, and it increases to 5.4% with PFHI (see Additional file 7). However, urban hospitalization rates are higher than rural rates, and hospitalization rates are also higher among those with higher incomes or higher social status, with or without insurance. A multiple logistic regression analysis with likelihood of hospitalization as the dependent variable showed that the likelihood of hospitalization increased with education, economic quintile and social status. The changes in the likelihood of hospitalization with sex and urban residence were minimal but significant. The likelihood of hospitalization did not change with insurance coverage (see Additional file 8).

## Discussion

Across nations, the emergence of universal health coverage as the dominant discourse on health policy has led to a renewed attention towards financial protection. In India as in many other developing nations, in the post-colonial period, the provision of free or subsidized care through government hospitals and health care facilities was the main form in which this was sought to be achieved. This changed in the 1990s with the introduction of health sector reforms linked to structural adjustment programmes. These reforms focused on limiting public services to a few select services that were chosen using the logic of least “dollar spent per DALY saved” and proposed leaving the rest of health care to the market [19]. Structural adjustment also resulted in a reduction in subsidies, introduction of user fees for cost recovery and restrictions on public sector employment, including that of nurses and doctors. These policies adversely affected the performance of health systems and equity in access to healthcare [20]. User fees are now accepted as highly exclusionary [21, 22], but in the 1990s, this was one of the main tenets of reform, and it still persists.

Low public funding has an adverse impact on both access to care and financial protection. Though lack of financial protection is a major cause for concern, the ideas that this requires a purchaser-provider split and that financial protection is most efficiently delivered through purchasing mechanisms need to be examined in context. Current evidence from India on the effectiveness of PFHIs in providing financial protection, even for hospitalization, to which they are currently largely limited, indicates a limited effectiveness of such programmes. Our results convey a new body of evidence to clearly demonstrate this point. Earlier studies by Karan and Selvaraj (2012) [23], Fan et al. (2011) [24] and Selvaraj et al. (2014) [25] that have used other data sets and different methods have also shown the limited effectiveness of PFHIs in providing financial protection.

In pluralistic health systems similar to those in India, it is essential for policy makers to be guided by periodic measurements of the levels of financial protection achieved and the contributions that different government interventions make towards achieving the criteria. We have used seven indicators that are relatively easy to generate from a household survey to measure the extent of financial protection or its converse, the extent of financial hardship suffered. These include the mean and median OOPE per hospitalization episode, the incidence of catastrophic health expenditures at 10% and 25% of annual consumption expenditure thresholds, the proportion of impoverishment due to health care costs and the proportion of hospitalization episodes where out-of-pocket medical expenditures were less than Rs 500 or less than Rs 1000. These seven indicators could easily be extended to measure financial protection for ambulatory care. The only reason that we did not do so was because we were measuring the effect of PFHIs in India, which in most schemes do not cover ambulatory care.

To guide policy choices, there is a need to develop a framework of measurement and analysis that is not only able to measure financial protection but also the level of equity with which such coverage is offered. Additionally, this framework should further attribute the effectiveness of such coverage or the lack of it to policy choices, particularly to the government purchasing care from private providers through insurance mechanisms, or the government providing access to free or subsidized care in government facilities and various combinations of the two [26].

All these seven indicators show that from the viewpoint of the care-seeker, insurance coverage by itself makes minimal difference to financial protection, and subsidized care from the public provider is more effective in reducing financial hardship. The median OOPE for hospitalization with a tax-funded public provider, even without PFHI coverage, is Rs 1451 (USD 24) and the incidence of CHE-10 is 16.1%. This is far lower than the median OOPE for hospitalization with a private provider in a household having PFHI coverage (Rs 10,120 or USD 169) and the incidence of CHE (60.0%). However, we note that when PFHI coverage complements the utilization of tax-funded public hospitalization, this allows for a further advantage in financial protection with median OOPE decreasing to Rs 950 (USD 16) per hospitalization and the incidence of CHE decreasing to 14.8%. In the lowest three quintiles, after matching for different potential confounders, the contribution that PFHIs make to the reduction in CHE-10 and CHE-25 measured by the PSM method is statistically insignificant.

We also note that utilization rates of hospital services, a proxy to access, also do not change with insurance once we adjust for other factors contributing to the levels of consumption of health care such as urban residence, higher income quintile, higher social group and higher educational status.

What is also a matter of concern is that unlike public provisioning, which was accessed more among lower income quintiles, more PFHI coverage occurs in higher quintiles, although by definition, this is a redistributive measure meant to provide increased access and financial protection for the poorest. The major reasons for the lower coverage of the poor and vulnerable population under PFHI compared to official projections have been attributed to exclusions at various levels, design failures and lack of awareness among the population [27]. Further higher PFHI coverage in the higher quintiles could be a reflection of the earlier social insurance schemes that cover government employees and organized workers.

However, all is not well with public provisioning. If nearly 32.3% of the poorest quintile in rural areas and 48.4% of the poorest in urban areas choose a private provider despite the high OOPE and CHE, then there are clearly other factors at work. This could be explained by unsatisfactory quality (42.7%), long waiting times (27.4%), distant facilities (11.6%), unavailable services (10.3%) and other causes (8.1%) [28, 29]. All of these are reflective of underperforming public health systems, which have been attributed to very low public health spending as well as many issues of design and implementation [30].

We note that the wide differential in OOPE between the poorest and richest quintile, even in the public sector, could indicate that there is an under-consumption of health care in the poorest quintiles. Utilization rates for hospitalization (used as a proxy for access) are also lower in poorer quintiles. It was in part to address these problems of access that PFHI were introduced. The fact that coverage with a PFHI, although it increased hospitalization rates, did not change the pattern of choice of provider indicates the need to look beyond financial barriers and quality of care for explaining the choice of provider.

All of this has policy implications for the choice between expanding the insurance schemes to cover a larger proportion of the population with a deeper benefit package, strengthening the provisioning of a more comprehensive package of free or subsidized care in public hospitals or a judicious combination of the two. It is true that unlike for the private sector, OOPE does not represent the entire costs of care of the public hospital since there is already a considerable amount invested by the government on the supply side. However, it does not follow that the costs of purchasing from the private sector are the same or lower than purchasing from the public sector. There is some evidence from state and national health accounts that the full costs of care are lower in a public hospital [31]. Moreover, the public hospital has a number of public functions to perform. The relative efficiencies of care provided versus care that is purchased would have to be studied separately. Our study indicates that PFHIs could play a useful source of additional financing for public hospitals that improves the effectiveness of financial protection in a manner more responsive to caseloads than that achieved by budget line item based financing. Potentially, such additional financing could be used to improve quality of care in public hospitals. However, whether the better strategic choice is to merely increase public financing of the public hospitals or not remains an open question.

The study also questions the effectiveness of currently existing models of PFHI in delivering financial protection. Admittedly, since current investment in PFHIs is small, population level effects on financial protection would be limited. However, our study is focused on hospitalisation episodes which is the mandate of PFHIs. Our concern is that if we are unable to see any effect on financial protection among those who have PFHI coverage and have been hospitalised, then we need to explore the reasons for this before we consider further expansion of purchasing from the private sector on such terms. This could be due to the lack of an appropriate regulatory regime and poor monitoring and governance leading to different forms of co-payment and manipulation, commonly referred to as ‘gaming the system’. If CHE rates due to hospitalization remain similar across income quintiles despite PFHI coverage (see Table 2), further research would be needed to explore the cause of this, including the possibility that the extent of co-payments is influenced by information asymmetries linked to the patient’s ability to pay and the provider’s ability to charge, rather than real needs and costs.

Our findings are in consonance with earlier studies from many nations, such as China, India and nations in Latin America [23, 32–34], and taken together, these results should provide caution to making road maps to universal health coverage that rely mainly on insurance schemes or purchasing from the private sector [21]. In the Indian context (and in many developing nations), regulation of the private sector is very weak, and there are high degrees of irrational care and conflict of interest situations [23, 24]. The institutional capacity for purchasing of care and contracting is also limited. Expanding to include ambulatory care into purchasing on such terms would have even higher risks. However, in parallel, there must be much more investment and effort made in strengthening public providers that are defined more broadly to include not-for-profit providers, as a vehicle not only for access but also for financial protection. This form of purchasing care could help in this regard.

This study has certain limitations. This survey collected information on all publicly funded health insurance schemes from various states and centres as grouped under one category, and two of these were earlier social insurance schemes that did not address the poor. We have addressed this limitation to an extent by analysing the poorer quintiles, but it would have been more robust if the questionnaire had been more specific regarding the type of PFHI. There are also inherent limitations in using only a cross-sectional study to comment on the impact of any intervention, although propensity score matching offers a more robust method to indicate the effect of an intervention in a cross-sectional study. Additionally, we note that although OOPE is a good measure of financial protection from the viewpoint of the beneficiary, to comment on the relative cost efficiency of subsidized public provision and PFHIs, we will also need to account for the entire costs of the respective strategies, which is beyond the scope of this paper.

## Conclusions

With respect to the problematic nature of measuring progress towards UHC, this paper illustrated the usefulness of this unique cross-sectional household survey to comment on the differential contribution that the main government strategies of purchasing and providing care make towards achieving this objective. India is a pluralistic health care system, and accurately determining the mix of purchasing and provisioning will remain one of the key issues that policy makers will face for some time to come.

## Additional files

Additional file 1: Different public funded health insurance (PFHI) programmes in India. This table shows the different PFHIs in India, including eligible beneficiaries, packages of services, sources of funding and covered population. This was obtained from different states’ websites and the World Bank Report, 2012. (XLSX 10 kb)

Additional file 2: Choice of Provider for hospitalization under different insurance schemes (as a percentage of all hospitalization episodes). This table was calculated by the authors using the NSS 71st Round unit level data. This table shows the extent of service provisioning under public and private facilities. (XLSX 9 kb)

Additional file 3: Average Out-of-pocket expenditure (OOPE) per hospitalization episode (the mean, in Rs.) across various socio-economic categories by type of insurance coverage. This table was calculated by the authors using the NSS 71st Round unit level data. This table shows OOPE across different equity indicators (sex, residence and social group). (XLSX 9 kb)

Additional file 4: Major source of finance for expenses incurred during hospitalization. This table was calculated by the authors using the NSS 71st Round unit level data. This table shows major sources of financing for households to meet their healthcare expenditure. (XLSX 10 kb)

Additional file 5: Second Major source of finance for expenses incurred during hospitalization. This table was calculated by the authors using the NSS 71st Round unit level data. This table shows the second major source of financing for households to meet the healthcare expenditure. (XLSX 11 kb)

Additional file 6: Average OOPE per hospitalization (in Rs, the median and the mean with 95% CI) by provider type and different insurance schemes. This table was calculated by the authors using the NSS 71st Round unit level data. This table shows OOPE in India in public and private provisioning under different insurance schemes. (XLSX 12 kb)

Additional file 7: Hospitalization rate (per 100 population) under different insurance schemes for various socio- economic stratifiers. This table was calculated by the authors using the NSS 71st Round unit level data. This table shows the hospitalization rate in India across different equity indicators. (XLSX 10 kb)

Additional file 8: Factors affecting likelihood of hospitalization in India. This table was calculated by the authors using the NSS 71st Round unit level data. It is based on a logistic regression and shows factors affecting hospitalization in India. (XLSX 9 kb)

## Abbreviations

ATT: Average Treatment on treated
BPL: Below poverty line
CHE: Catastrophic health expenditure
CHE-10: Proportion of households in a population who face catastrophic health expenditure computed using the threshold of 10% of usual annual consumption expenditure
CHE-25: Proportion of households in a population who face catastrophic health expenditure computed using the threshold of 25% of usual annual consumption expenditure
DALY: Disability-adjusted life year
ESI: Employees’ state insurance
MPCE: Monthly per capita expenditure
NSS: National sample survey
NSSO: National sample survey office
OOPE: Out-of-pocket expenditure
PFHI: Public Funded Health Insurance
PSM: Propensity score matching
RSBY: Rashtriya Swasthya Bima Yojana
UHC: Universal Health Coverage
UMPCE: Usual monthly per capita expenditure
